# The PK–PD Relationship and Resistance Development of Danofloxacin against *Mycoplasma gallisepticum* in An *In Vivo* Infection Model

**DOI:** 10.3389/fmicb.2017.00926

**Published:** 2017-05-30

**Authors:** Nan Zhang, Yuzhi Wu, Zilong Huang, Lihua Yao, Longfei Zhang, Qinren Cai, Xiangguang Shen, Hongxia Jiang, Huanzhong Ding

**Affiliations:** ^1^Guangdong Key Laboratory for Veterinary Drug Development and Safety Evaluation, South China Agricultural UniversityGuangzhou, China; ^2^Technical Center for Inspection and Quarantine, Zhuhai Entry-Exit Inspection and Quarantine BureauZhuhai, China

**Keywords:** *Mycoplasma gallisepticum*, danofloxacin, *in vivo* infection model, mutation, killing

## Abstract

*Mycoplasma gallisepticum* is the causative agent of chronic respiratory disease (CRD), a prevalent disease of poultry, which is responsible for significant economic losses in farms. Although several antimicrobial agents are currently recommended for the treatment and prevention of *M. gallisepticum* infections, investigations of *M. gallisepticum* have been hampered by their fastidious growth requirements and slow growth rate. As such, little work has been conducted concerning the PK/PD relationship and mechanisms of antibiotic resistance between antimicrobials against *M. gallisepticum*. In the present study, danofloxacin was orally administrated to the infected chickens once daily for 3 days by an established *in vivo M. gallisepticum* infection model. Not only the concentrations of danofloxacin in plasma and lung tissues were analyzed, but also the counting of viable cells and changes in antimicrobial susceptibility in air sac and lung were determined. The PK and PD data were fitted by WinNonlin to evaluate the PK/PD interactions of danofloxacin against *M. gallisepticum*. PCR amplification of quinolone resistance-determining regions (QRDRs) and DNA sequencing were performed to identify point mutations in *gyrA, gyrB, parC*, and *parE* of the selected resistant mutant strains. In addition, susceptibility of enrofloxacin, ofloxacin, levofloxacin, gatifloxacin, and norfloxacin against these mutant strains were also determined. The PK profiles indicated that danofloxacin concentration in the lung tissues was higher than plasma. Mycoplasmacidal activity was achieved when infected chickens were exposed to danofloxacin at the dose group above 2.5 mg/kg. The ratios of AUC_24_/MIC (the area under the concentration-time curve over 24 h divided by the MIC) for 2 log_10_ (CFU) and 3 log_10_ (CFU) reduction were 31.97 and 97.98 L h/kg, respectively. Substitutions of Ser-83→Arg or Glu-87→Gly in *gyrA*; Glu-84→Lys in *parC* were observed in the resistant mutant strains that were selected from the dose group of 1 and 2.5 mg/kg. MICs of danofloxacin, enrofloxacin, ofloxacin, levofloxacin, gatifloxacin, and norfloxacin against the resistant mutant strains with a single mutation in position-83 were higher than that with a single mutation in position-87. These findings suggested that danofloxacin may be therapeutically effective to treat *M. gallisepticum* infection in chickens if administered at a dosage of 5.5 mg/kg once daily for 3 days.

## Introduction

*Mycoplasma gallisepticum* is a primary pathogen that is responsible for chronic respiratory disease (CRD) in chickens as well as sinusitis in turkeys. The primary signs of *M. gallisepticum* infections include nasal discharge, keratoconjunctivitis, air sacculitis, and depression (Levisohn and Kleven, 2000). *M. gallisepticum* infections cause considerable economic losses in poultry industry as a result of increased embryo mortality, reduced egg production and decreased weight gain (Kleven, 1990). *M. gallisepticum* can be transmitted horizontally from infected individuals to healthy individuals by aerosol route or vertically via eggs. Control of *M. gallisepticum* infections with antimicrobial therapy is usually effective and economically feasible. *M. gallisepticum* is susceptible to some antibacterial agents *in vitro*, such as quinolones, tetracyclines, macrolides, and pleuromutilins. However, increasing drug resistance in *M. gallisepticum* has been discovered in the past decades (Pakpinyo and Sasipreeyajan, 2007; Gerchman et al., 2008). Danofloxacin, one of the synthetic quinolones, has play a significant role in treatment of *M. gallisepticum* infections (Jordan et al., 1993).

*Mycoplasma gallisepticum* should be cultivated on specially formulated media, for the reason that *Mycoplasma* is dependent on outside sources of precursor molecules for macromolecular syntheses (Razin and Tully, 1970; Greenberg-Ofrath et al., 1993). Moreover, viable count estimation (CFU determination) is difficult by the strict nutritional conditions for growth. Therefore, there were a few reports concerning the PK/PD relationship and mechanisms of antibiotic resistance between antimicrobials against *M. gallisepticum*. In the past years, *in vitro* models, *ex vivo* models, and *in vivo* models have been established to evaluate the characteristics of antibacterial agents against *M. gallisepticum* (Xiao et al., 2015a,b; Zhang et al., 2016). Antimicrobial chemotherapy with an optimal dose-response outcome may be based on the relationship of PK/PD *in vivo* model (Vinks et al., 2014).

Most of the published articles indicated that the substitutions in quinolone resistance-determining region (QRDR) were generally induced by point mutations in topoisomerase and gyrase. The mutations in QRDR of *gyrA, gyrB, parC*, and *parE* were correlated with the development of quinolone resistance in *M. gallisepticum* (Reinhardt et al., 2002a,b; Lysnyansky et al., 2008, 2012). After different dosage regimens, the resistant mutant strains were selected from the tissues. Then mutations in the QRDR of *gyrA, gyrB, parC*, and *parE* were identified in the current study. The purpose of this study is to characterize the PK–PD relationship and resistance development of danofloxacin against *M. gallisepticum* in an *in vivo* infection model where viable cells count estimation was performed by isolating *M. gallisepticum* from the infected chickens.

## Materials and Methods

### Organisms and Chemicals

*Mycoplasma gallisepticum* strain S6 was obtained from the Chinese Veterinary Microorganism Culture Collection Center (Beijing, China). It was initially isolated from Nippon Institute for biological science in 1958 (Adler et al., 1958). Moreover, S6 is a recognized common clinical isolate in China. Danofloxacin mesylate standard (>99%), enrofloxacin standard (98%), ofloxacin standard (97%), levofloxacin standard (97%), gatifloxacin standard (99%), norfloxacin standard (98%), and penicillin sodium were kindly supplied by Guangdong Dahuanong Animal Health Products.

### Media

*Mycoplasma gallisepticum* growth medium base was provided by Qingdao Hope Biological Technology. Sterile pig serum was purchased from Guangzhou Ruite Biological Technology. Nicotinamide adenine dinucleotide (NADH) and cysteine were obtained from Guangzhou Prob Information Technology. The *M. gallisepticum* growth medium base was dissolved with water and autoclaved at 115°C for 30 min. NADH and Cysteine were sterilized by 0.22 μm filtration and then added to the *M. gallisepticum* medium base at a final concentration of 0.2%. Inactivated pig serum was further supplemented at the final concentration of 10%. Eighty (80) million IU of penicillin was also added into the prepared medium. Finally, its pH was adjusted to 7.8 ± 0.1 with 1 mol/L NaOH and kept at 4°C for use within 2 weeks. Solid medium was prepared by adding 1% agar into the growth medium. The culture of *M. gallisepticum* was diluted with the growth medium by 10-fold serial dilutions, then 10 μL of each *M. gallisepticum* dilutions were transferred to the surface of *M. gallisepticum* agar plates. After being sealed with gas permeable film, the plates were incubated for at least 7 days in a 37°C, 5% CO_2_ humidified incubator and colonies of *M. gallisepticum* were counted by the inverted microscope (Leica Microsystems DM1L, Germany).

### Susceptibility Determination

The standard of 0.33 g danofloxacin mesylate, 0.261 g enrofloxacin, 0.264 g ofloxacin, 0.264 g levofloxacin, 0.259 g gatifloxacin, and 0.261 g norfloxacin were dissolved in 100 mL sterile distilled water to get the initial concentration of 2560 mg/L, respectively. The final drug concentration of agar plates ranged from 0.019 to 38.40 mg/L in double dilution.

The *in vitro* susceptibility of danofloxacin, enrofloxacin, ofloxacin, levofloxacin, gatifloxacin, and norfloxacin against *M. gallisepticum* strain S6 as well as danofloxacin resistant mutant strains isolated from chickens were determined by the agar dilution methods as described by Hannan (2000). The single colony was selected and cultured in drug-free growth medium for 5 days. When color of the medium changed from red to orange or yellow (*M. gallisepticum* was in logarithmic phase), the concentration of *M. gallisepticum* was 10^8^ CFU/mL. The culture was diluted to 10^7^ CFU/mL by the blank growth medium. Then a 10 μL sample of *M. gallisepticum* culture (10^7^ CFU/mL) was inoculated onto agar plates containing two-fold serial dilutions of quinolones. Growth control (*M. gallisepticum* inoculum without quinolones) and sterility control (blank medium) were also included in the MIC determination. *E. coli* ATCC 25922 was used for quality control strain. The MIC was determined as the minimal concentration of antibacterial agent that resulted in no growth on the agar plate after 7 days.

### Animals and Inoculation

All *in vivo* experiments were approved by the animal research committees of South China Agriculture University Animal Ethics Committee (Approve number: 2015 A027). One-day-old chickens were provided from Guangdong Academy of Agricultural Sciences (Guangzhou, Guangdong, China). The chickens were *M. gallisepticum* free and fed with antimicrobial-free feed.

In preliminary experiments, the chickens were inoculated with a 0.2-mL aliquot solution containing 10^7^, 10^8^, and 10^9^ CFU/mL of *M. gallisepticum* (twice a day) via intratracheal injection for 3 days (*n* = 30/group). After inoculation, the infected chickens were anatomized. The signs of disease and pathological changes were observed to validate the success of the experimental infection. *M. gallisepticum* ELISA kit were purchased from Shenzhen Lvshiyuan Biotechnology Technology. Serum was also collected from the infected chickens and the antibody of *M. gallisepticum* was measured by the commercially available ELISA kits according to the supplier’s instructions. The optical density at 450 nm (OD_450_) was recorded using a universal Microplate Reader ELx800 (Bio-Tek Instruments, United States). Besides, the load of *M. gallisepticum* in air sac and lung were monitored to confirm infection. To ensure the accuracy of the results, this experimentation was performed for three times. All chickens were housed in the same animal room and received identical daily care. Moreover, the feeding environment was controlled rigidly in order to prevent other *M. gallisepticum* infection.

Evaluation of the *M. gallisepticum* infection model was mainly based on the clinical signs and colonization of *M. gallisepticum*. The infected chicken exhibit mostly respiratory presentations including ocular and nasal discharge, sneezing, cough, breathing difficulty/mouth breathing, and moist rales. Catarrhal inflammation (sinuses, trachea, and bronchi), the thickened and opaque of air sacs and lung enlargement were found in most of *M. gallisepticum* infected chickens. Additionally, *M. gallisepticum* specific antibody was observed in the infected chickens.

A total of 1140 chickens were randomly divided into five groups. (1) A group of 840 chickens were used to study the pharmacokinetics of danofloxacin in infected chickens; (2) A group of 210 chickens were used to study the pharmacodynamics of danofloxacin in infected chickens; (3) 40 chickens were used as growth control group to observe the load of *M. gallisepticum* in infected chickens; (4) 40 chickens were used as negative control group to observe the load of *M. gallisepticum* in healthy chickens; (5) The remaining 10 health chickens were used as blank control to obtain blank plasma and lung tissue. The chickens in growth and negative control group were administrated by oral gavage with 0.85% NaCl once daily for 3 days. The chickens in blank group were untreated and the blank samples (plasma and lung tissues) were collected before the experiment.

### Danofloxacin Pharmacokinetics in *In Vivo* Infection Model

The chickens were inoculated with a 0.2-mL aliquot solution containing 10^9^ CFU/mL of *M. gallisepticum* (twice a day) via intratracheal injection for 3 days. Groups of infected chickens were orally administrated by oral gavage with danofloxacin at a dose of 1, 2.5, 5, 10, or 20 mg/kg once daily for 3 days, and they were euthanized at 0.25, 0.5, 1, 2, 4, 6, 8, 12, 24, 25, 26, 28, 32, 36, 48, 49, 50, 52, 56, 60, and 72 h after the first oral gavage of danofloxacin. Blood and lung tissues were collected from eight chickens at each sampling time point per treatment group. Blood samples were centrifuged at 3,000 ×*g* for 10 min at 4°C, and then plasma was collected. The samples of plasma and lung tissues were stored at -20 until analyzed by HPLC within 2 weeks.

Danofloxacin concentrations in plasma and lung tissues were determined by high performance liquid chromatography with fluorescence detection (HPLC-FD) (Agilent Technologies, United States). The HPLC was equipped with an Agilent TC-C18 column (250 mm × 4.6 mm, 5 μm) using a mobile phase of triethylamine phosphate (pH 2.4): acetonitrile (19:81, v/v) and a flow rate of 0.8 mL/min. Injection volume was 20 μL. A calibration curve was established with seven danofloxacin concentrations (0.001–0.1 μg/mL).

Plasma sample (0.5 mL) was added to 0. 5 mL of acetonitrile, vortexed for 1 min and then incubated in a 45°C water bath for 10 min to precipitate proteins. After centrifugation at 10,000 ×*g* at 4°C for 5 min, 0.5 mL of supernatant were transferred to a centrifuge tube containing 0.5 mL of ultra-pure water. Finally, the mixture was vortexed for 30 s and filtered through a 0.22 μm syringe filter prior to HPLC analysis.

Lung tissue sample (0.15 g) was homogenized and then 1.5 mL of Mcllvaine–Na_2_EDTA buffer (PH 4.0 ± 0.5) was added to extract danofloxacin. The samples were vortexed for 1 min and incubated in a 45°C water bath for 10 min to precipitate proteins. After centrifugation at 10,000 ×*g* at 4°C for 5 min, the supernatant was filtered through a 0.22 μm syringe filter prior to HPLC analysis.

Pharmacokinetic parameters, including elimination half-life (*t*_1/2_), the area under the concentration-time curve over 24 h (AUC_24_), and maximum concentration of drug in samples (*C*_max_), the time of peak concentration (*T*_max_) were calculated by the non-compartmental model using the WinNonlin software (version 6.1, Pharsight).

### Statistical Analysis

*T*-test was used for statistical analysis. The differences of morbidity and mortality of chickens in different inoculum sizes as well as *T*_max_ and *t*_1/2_ of danofloxacin in plasma and lung were calculated. *P* < 0.05 was considered to be statistically significant.

### Efficacy of Danofloxacin against *M. gallisepticum* in Chicken Infection Model and Changes in Susceptibility

The chickens were inoculated with a 0.2-mL aliquot solution containing 10^9^ CFU/mL of *M. gallisepticum* (twice a day) via intratracheal injection for 3 days. Then the infected chickens were administrated by oral gavage with danofloxacin at the dose of 1, 2.5, 5, 7.5, 10, 15, or 20 mg/kg once daily for 3 days. At 24 h after each drug administration, the chickens were euthanized. The air sacs and lung tissues were aseptically collected and homogenized in 1 mL of medium. Viable cell numbers were determined via 10-fold serial dilutions and plating 10 μL of each diluted culture onto agar plates without danofloxacin. The samples (air sacs and lung tissues) were obtained from 10 chickens at each sampling time point per treatment group. Values for reduction less than 3 log_10_ CFU denoted mycoplasmastasis activity; whereas values more than or equal to 3 log_10_ CFU indicated mycoplasmacidal.

When the chickens were euthanized at 72 h following the initial administration, the samples were homogenized and vortexed in 1 mL of medium, then 50 μL samples were incubated in 10 mL drug-free growth medium. When color of the medium changed to yellow (*M. gallisepticum* was cultured in logarithmic phase), the MIC was determined by the agar dilution method using the agar plates containing serial danofloxacin concentrations. A two-fold increase of danofloxacin concentrations were used in susceptibility determination, which ranged from 1XMIC to 128XMIC. The resistant mutants could be selected when danofloxacin concentrations of the agar plates were above MIC.

### PK–PD Analysis

Pharmacokinetic profiles of danofloxacin in lung tissues were analyzed by non-compartmental model using WinNonlin software (version 6.1; Pharsight, CA, United States). The pharmacokinetic–pharmacodynamic index of AUC_24_/MIC was calculated using *in vitro* MIC values and PK parameters derived from danofloxacin concentration in lung tissues samples. Effectiveness of danofloxacin was reflected as the reduction in viable cells of *M. gallisepticum* in lung tissues after each treatment compared to the control group. The PK/PD analysis was performed by using a sigmoid maximum effect (*E*_max_) model. This model is described by the following equation:

$$E=E_{\max}-(E_{\max}-E_{0})\times C_{e}^{N}/(EC_{50}^{N}+C_{e}^{N})$$

Where *E* is the antibacterial effect that was assessed as the reduction in log_10_ CFU/lung after each administration of danofloxacin, compared to the log_10_ CFU/lung in untreated control group; *E*_max_ is the reduction in log_10_ CFU/lung for the untreated control chickens; *E*_0_ is the maximum reduction after administration that represents the maximum antibacterial effect; *C*_e_ is the AUC_24_/MIC parameter; EC_50_ is the AUC_24_/MIC value required to achieve 50% of the maximal antibacterial effect; and *N* is the Hill coefficient that describes the steepness of the AUC_24_/MIC and effect curve.

### Dosage Calculation

For the purpose of deducing a optimal regimen, the dose required for different magnitudes of efficiency is provided by the following equation described by Toutain et al. (2007):

$$Dose\,\;(per\,\;day)={\left(AUC/MIC\right)}_{break\,\;po\,\mathrm{int}}\times {MIC}_{90}\times {CL}_{per\,\;hour}/\left(f_{u}\times F\right)$$

Where dose (per day) is the optimal dose (mg/kg⋅bw); AUC/MIC is the targeted end point for the desired effect (L⋅h/kg); MIC_90_ is the 90% of the MIC distribution (mg/L); and clearance is the lung clearance expressed as kg/kg/h (CL_perhour_ was 0.373 kg/kg/h). *f*_u_ is the free drug fraction, and F is the bioavailability.

### PCR Amplification of Quinolone Resistance-Determining Regions (QRDRs) and DNA Sequencing

The components used for PCR (Polymerase Chain Reaction) were obtained from Takara Bio. The resistant mutant strains were conserved firstly when selected from the agar plates containing serial danofloxacin concentrations. In this stage, three individual mutant strain of each sample with increased MICs were selected and conserved. Single colony of these mutants was passaged for five generations independently. The mutations in *gyrA, gyrB, parC*, and *parE* were identified before and after their respective passages to determine whether the mutations could be inherited steadily. DNA extraction of the selected mutants and PCR amplification were performed using a previously described protocol (Ley et al., 1997). *gyrA, gyrB, parC*, and *parE* were amplified by the specific primers designed from strain S6 of *M. gallisepticum* (**Table 1**). Amplify reaction was achieved as following: PCR mixture was consisted of 2.5 μL 10 × E_X_ Taq buffer (Mg^2+^ plus), 2.5 μL dNTP, 0.5 μL primer F (10 μmol/mL), 0.5 μL primer R (10 μmol/mL), 1 μL of extracted *M. gallisepticum* DNA, 0.25 μL E_X_ Taq and 17.75 μL distilled water. Positive control (*M. gallisepticum* strain S6) and blank control (distilled water) were also performed concurrently for each reaction. The amplification conditions were at 94°C for 5 min, 94°C for 45 s, 55°C for 45 s, 72°C for 1 min for 30 cycles. The final extension cycles were at 72°C for 5 min. After amplification, the sequencing reaction was performed by Beijing Genomics Institute using Sanger sequencing.

**Table 1:** Nucleotide sequences of the primers used for PCR.

Primer	Sequence
*gyrA*	F 5′-TATGG TGCTTACACT TCAG-3′
	R 5′-CTACGGCAAT ACCACTTG-3′
*gyrB*	F 5′-TGACGGTAAGATTAGCAAAG-3′
	R 5′-ACATCAGCATCGGTCATGA-3′
*parC*	F 5′-ATGGATAAGAAAAAGGTATTTCAAAAAG-3′
	R 5′-TTAACGAGTAAGTTAGGTAATAAACTAGGTAAGAT-3′
*parE*	F 5′-GGTATCAAATTACAACGAAAAAC-3′
	R 5′-CCACCATCTTGGTAGATCGA-3′

## Results

### Susceptibility Determination

The MICs of danofloxacin, enrofloxacin, ofloxacin, levofloxacin, gatifloxacin, norfloxacin against *M. gallisepticum* S6 parental and its mutant strains are shown in **Table 2**. For culture 1, mutant strains selected from the dose group of 1 mg/kg had a (Ser-83→Arg) substitution at the position corresponding to amino acid 83 in *gyrA* of *E. coli*. For culture 2, mutant strains selected from the dose group of 1 mg/kg carried a (Glu-87→Gly) substitution at the position of 87 in *gyrA* of *E. coli*. For culture 3, mutant strains selected from the dose group of 2.5 mg/kg had a (Ser-83→Arg) substitution at the position corresponding to amino acid 83 in *gyrA* of *E. coli*. For culture 4, mutant strains selected from the dose group of 2.5 mg/kg and had a (Ser-83→Arg) substitution at the position corresponding to amino acid 83 in *gyrA* of *E. coli* and an additional (Glu-84→Lys) substitution at the position corresponding to amino acid 84 in *parC* of *E. coli*. Gatifloxacin was found to exhibit marginally superior activity against *M. gallisepticum* and its MIC was 0.038 mg/L for S6; 0.6 mg/L for resistant mutant culture 1, 3, and 4; 0.15 mg/L for mutant culture 2, respectively. The mutant culture 1, 3, 4 harboring a (Ser-83→Arg) substitution in *gyrA* had an increased MIC to danofloxacin, enrofloxacin, ofloxacin, levofloxacin, gatifloxacin, norfloxacin. This value was 4- to 16-fold higher than culture 2 containing a (Glu-87→Gly) substitution in *gyrA*.

**Table 2:** Antibiotic susceptibilities of the parental vs. mutant strains.

Strains	MIC (mg/L)					
	Danofloxacin	Enrofloxacin	Ofloxacin	Levofloxacin	Gatifloxacin	Norfloxacin
S6	0.15	0.15	0.3	0.15	0.038	0.6
Culture 1	4.8	4.8	9.6	9.6	0.6	19.2
Culture 2	0.6	0.6	1.2	0.6	0.15	2.4
Culture 3	4.8	4.8	9.6	9.6	0.6	19.2
Culture 4	4.8	4.8	9.6	9.6	0.6	19.2

*Culture 1: The mutant strains that selected from the dose group of 1 mg/kg and had a (Ser-83→Arg) substitution at the position corresponding to amino acid 83 in *gyrA* of *E. coli*. Culture 2: The mutant strains that selected from the dose group of 1 mg/kg and had a (Glu-87→Gly) substitution at the position of 87 in *gyrA* of *E. coli*. Culture 3: The mutant strains that selected from the dose group of 2.5 mg/kg and had a (Ser-83→Arg) substitution at the position corresponding to amino acid 83 in *gyrA* of *E. coli*. Culture 4: The mutant strains that selected from the dose group of 2.5 mg/kg and had a (Ser-83→Arg) substitution at the position corresponding to amino acid 83 in *gyrA* of *E. coli* and an additional (Glu-84→Lys) substitution at the position corresponding to amino acid 84 in *parC* of *E. coli*.*

### *M. gallisepticum* Infection Model

The inoculum size, morbidity, mortality, *M. gallisepticum* load in air sac and lung are presented in **Table 3**. When the inoculum size was 7.26 log_10_ CFU/mL, the morbidity and mortality were far lower than that of 8.33 and 9.31 log_10_ CFU/mL. There were no significant differences in morbidity (*p* = 0.12) and mortality (*p* = 0.58) between the inoculum size of 8.33 and 9.31 log_10_ CFU/mL, but different number of colonies was observed in air sac and lung. *M. gallisepticum* load in air sac and lung appeared to always depend on the inoculum size. As the inoculum size increased, the viable count of *M. gallisepticum* in air sac and lung increased evidently. When the inoculum size was 9.31 log_10_ CFU/mL, *M. gallisepticum* load in air sac and lung were approximately 0.5 log_10_ CFU/mL more than that of 8.33 log_10_ CFU/mL. Based on the morbidity, mortality, *M. gallisepticum* load in air sac and lung, the best inoculum size for the infection model was 10^9^ CFU/mL. Neither clinical signs of disease nor antibody of *M. gallisepticum* were observed in negative control group. When the viable cells of air sac and lung from the negative control group were quantitated, no *M. gallisepticum* was found from the agar plates.

**Table 3:** The inoculum size, morbidity, mortality, *M. gallisepticum* load in air sac and lung.

Inoculum size (Log_10_ CFU/mL)	Morbidity rate (%)	Mortality rate (%)	MG load in air sac (Log_10_ CFU/air sac)	MG load in lung (Log_10_ CFU/lung)
7.26 ± 0.07	75.56 ± 3.85	7.78 ± 3.85	4.34 ± 0.56	4.28 ± 0.50
8.33 ± 0.12	94.44 ± 1.92	15.56 ± 5.09	5.61 ± 0.09	5.55 ± 0.10
9.31 ± 0.10	98.89 ± 1.92	17.78 ± 3.85	6.17 ± 0.30	6.09 ± 0.33

*Values are mean ± SD.*

### PK Profiles of Danofloxacin in Infection Model

**FIGURE 1:**
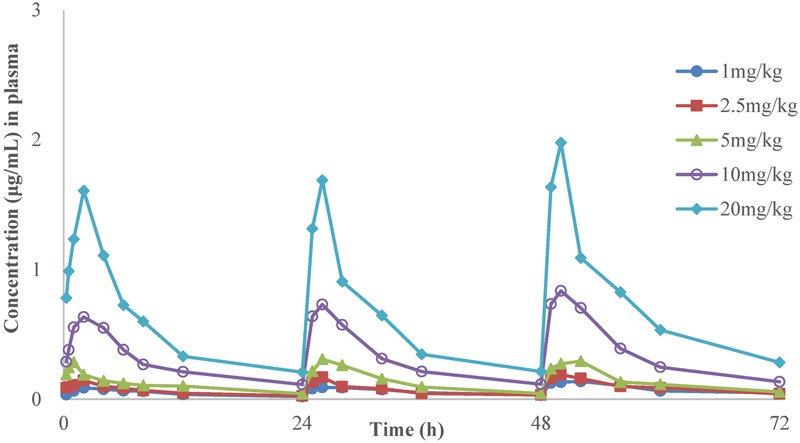
**The time-concentration curves of danofloxacin in plasma after three oral administrations doses of 1, 2.5, 5, 10, and 20 mg/kg in *M. gallisepticum* infection model (*n* = 8/time point)**.

**FIGURE 2:**
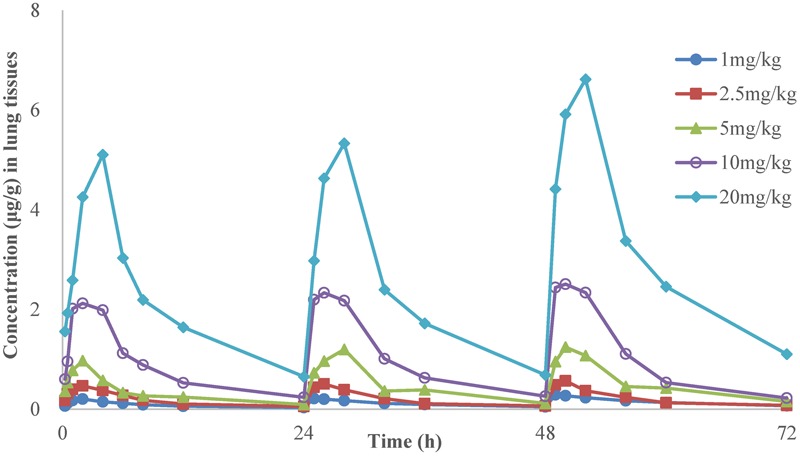
**The time-concentration curves of danofloxacin in lung tissues after three oral administrations doses of 1, 2.5, 5, 10, and 20 mg/kg in *M. gallisepticum* infection model (*n* = 8/time point)**.

The time-concentration curves of danofloxacin in plasma and lung tissues after three oral administrations at doses of 1, 2.5, 5, 10, and 20 mg/kg are shown in **Figures 1, 2**, respectively. The main PK parameters obtained from plasma and lung tissues are presented in **Tables 4, 5**, respectively. The mean *T*_max_ was 2.20 ± 0.3 h in plasma and 2.40 ± 1.01 h in lung tissues for all five different doses. There were no significant differences in *T*_max_ between lung and plasma (*p* = 0.68). The mean half-life (*T*_1/2_) was 11.5 ± 1.00 h in plasma and 9.2 ± 1.34 h in lung tissues. There were significant differences in *t*_1/2_ between lung and plasma (*p* = 0.0092). Besides, the PK parameters were dose-dependent, where the AUC_24_ values for the escalating doses ranged from 1.49 to 14.54 (μg⋅h/mL) in plasma and 2.72 to 58.58 (μg⋅h/g) in lung tissues, respectively. A significant correlation (*R*^2^> 0.96) was found between dose and AUC_24_ according to the linear relationship for each day after three oral administrations doses ranged from 1 to 20 mg/kg.

**Table 4:** Pharmacokinetic parameters of danofloxacin in plasma following three oral administrations of various doses in *M. gallisepticum* infected chickens.

Dose (mg/kg)	*T*_max_ (h)	*C*_max_ (μg/ml)	*t*_1/2_ (h)	AUC_24_ (μg⋅h/ml)
1	2.67 ± 1.15	0.11 ± 0.03	12.85 ± 1.78	1.49 ± 0.44
2	2.00 ± 0.00	0.17 ± 0.02	11.97 ± 1.05	1.76 ± 0.45
5	2.33 ± 1.53	0.30 ± 0.01	11.28 ± 1.84	2.99 ± 0.39
10	2.00 ± 0.00	0.73 ± 0.10	11.26 ± 0.30	7.39 ± 0.95
20	2.00 ± 0.00	1.76 ± 0.19	10.15 ± 0.31	14.54 ± 2.26

*Values are mean ± SD. *T*_*max*_, the time of peak concentration; *C*_*max*_, maximum concentration; *t*_*1/2*_, elimination half-life; AUC_*24*_, 24-h area under concentration-time curve.*

**Table 5:** Pharmacokinetic parameters of danofloxacin in lung tissues following three oral administrations of various doses in *M. gallisepticum* infected chickens.

Dose (mg/kg)	*T*_max_ (h)	*C*_max_ (μg/ml)	*t*_1/2_ (h)	AUC_24_ (μg⋅0h/ml)
1	1.33 ± 0.58	0.24 ± 0.05	10.88 ± 0.44	2.72 ± 0.80
2	2.00 ± 0.00	0.51 ± 0.05	8.33 ± 0.30	4.56 ± 0.36
5	2.67 ± 1.15	1.14 ± 0.15	9.69 ± 0.72	10.26 ± 2.42
10	2.00 ± 0.00	2.32 ± 0.19	8.05 ± 0.57	21.99 ± 1.92
20	4.00 ± 0.00	5.69 ± 0.81	9.04 ± 0.86	58.58 ± 12.61

*Values are mean ± SD. *T*_*max*_, the time of peak concentration; *C*_*max*_, maximum concentration; *t*_*1/2*_, elimination half-life; AUC_*24*_, 24-h area under concentration-time curve.*

### PD of Danofloxacin in Chicken Infection Model and Susceptibility Changes

**FIGURE 3:**
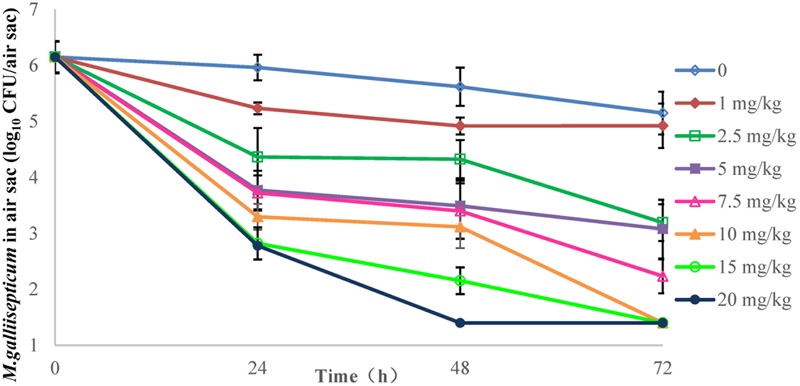
**Viable counts of *M. gallisepticum* in air sac after three oral administrations of danofloxacin (*n* = 10/time point)**.

**FIGURE 4:**
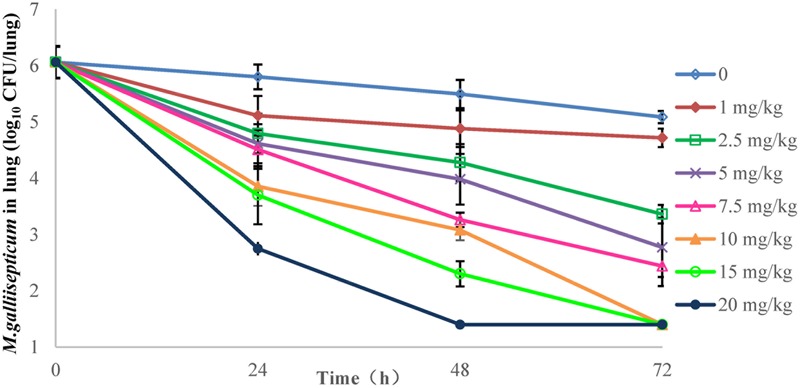
**Viable counts of *M. gallisepticum* in lung tissues after three oral administrations of danofloxacin (*n* = 10/time point)**.

The effects of danofloxacin against *M. gallisepticum* in air sac and lung tissues with different regimens are shown in **Figures 3, 4**, respectively. As danofloxacin concentration increased, antimycoplasmal activity gradually increased. Both in air sac and lung, mycoplasmacidal activity was observed when *M. gallisepticum* was exposed to concentration of danofloxacin at a dose above 2.5 mg/kg. When the dose was at 1 mg/kg, the MICs increased to 0.6 mg/L in 4 out of 20 samples and the MICs increased to 4.8 mg/L in the remaining 16 samples. The mutants were selected from the agar plates whose danofloxacin concentrations were 0.3 or 2.4 mg/L, respectively. When the dose was 2.5 mg/kg, a decreased susceptibility was observed in all of the samples, and the MICs increased prominently to 4.8 mg/L. The mutants were selected from the agar plates whose danofloxacin concentration was 2.4 mg/L. Three individual mutant strain of each sample with increased MICs were passaged for five times. When the doses were at 5 and 7.5 mg/kg, the MIC of danofloxacin against the survival cells was determined. The danofloxacin MIC of the parental strain *M. gallisepticum* was 0.15 and no mutants were observed in the agar plate containing danofloxacin ≥0.15 mg/L. When the doses were at 10, 15, and 20 mg/kg, no survival cells were recovered in the treatment groups at 72 h.

### PK and PD Analysis

**FIGURE 5:**
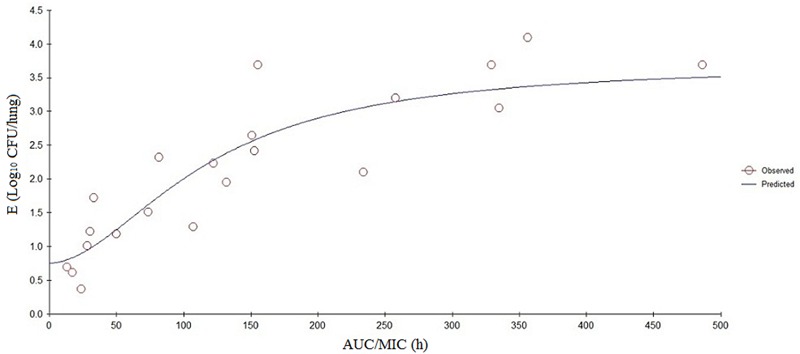
**Sigmoid *E*_max_ relationships between antimycoplasmal effect (*E*, log_10_ CFU/lung) and *in vivo* AUC_24_/MIC ratio against *M. gallisepticum* in the lung tissues of chickens**.

The PK/PD parameters (AUC_24_/MIC) derived from *in vivo* infection model and anti-mycoplasmal effects are presented in **Figure 5**. The PK–PD parameters are described in **Table 6**. AUC_24_/MIC correlated well with anti-mycoplasmal efficacy (*R*^2^ = 0.8901). The dose–response relationships with danofloxacin against *M. gallisepticum* were evaluated using Sigmoid *E*_max_ model. The ratios of AUC_24_/MIC for 2 log_10_ CFU/lung reduction and 3 log_10_ CFU/lung reduction were 31.97 and 97.98 L⋅h/kg, respectively.

**Table 6:** PK–PD analysis of danofloxacin in *M. gallisepticum* infection model.

Parameter	Value
*E*_max_ (log_10_ CFU/ml)	0.72 ± 0.50
*E*_0_ (log_10_ CFU/ml)	3.70 ± 0.84
EC_50_ (*h*)	115.02 ± 39.22
Slope (*N*)	1.77 ± 1.34

*Values are mean ± SD. *E*_*max*_, the reduction in log CFU/lung for the untreated control chicken. *E*_*0*_, the maximum reduction after administration that represents the maximum antibacterial effect. EC_*50*_, the AUC_*24*_/MIC value required to achieve 50% of the maximal antibacterial effect. *N*, the Hill coefficient which describes the steepness of the AUC/MIC and effect curve.*

### Dosage Calculation

Since there were not enough danofloxacin MIC data available to provide an estimate of the MIC_90_, the MIC of danofloxacin against *M. gallisepticum* strain S6 was substituted as a projected MIC_90_. The bioavailability had been taken into account, owing to the extravascular route of administration, and the free-drug fraction was not required for using PD data generated *in vivo*. A dosage of 5.5 mg/kg once daily for 3 days was calculated to be capable of a reduction of 3 log_10_ CFU/lung.

### Characterization of Point Mutations in DNA Gyrase and Topoisomerase IV Involved in Danofloxacin Resistance of *Mycoplasma gallisepticum* Mutants Recovered from *In Vivo*

The mutation in *gyrA, gyrB, parC*, and *parE* of the mutant strains obtained before and after the passages were the same. The mutant strains with increased MICs (0.6 and 4.8 mg/L) were selected from the two dosage of 1 and 2.5 mg/kg. Six colonies from each mutant strain were screened for each sample before and after the passage, and 20 samples (10 air sacs and 10 lung tissues) were collected from each dosage. Mutations in QRDR target genes are shown in **Table 7**. When the dose was 1 mg/kg, 16 samples had a (Ser-83→Arg) substitution at the position corresponding to amino acid 83 in *gyrA* of *E. coli*; and 4 samples had a (Glu-87→Gly) substitution at the position of 87 in *gyrA* of *E. coli*. When the drug was 2.5 mg/kg, 20 samples had a (Ser-83→Arg) substitution at the position corresponding to amino acid 83 in *gyrA* of *E. coli*; and 7 samples had an additional (Glu-84→Lys) substitution at the position corresponding to amino acid 84 in *parC* of *E. coli.* None of the mutants possessed any base changes in *gyrB* and *parE* (**Table 7**). No point mutations in QRDR targets were found in positive control group (*M. gallisepticum* strain S6).

**Table 7:** Amino acid changes in *gyrA, gyrB, parC*, and *parE* in danofloxacin-resistant strains.

Dose (mg/kg)	Strains	MIC (mg/L)	Mutations in QRDR target genes^a^			
			*gyrA*	*gyrB*	*parC*	*parE*
1	1 (*N* = 16)	4.8	S83R	–	–	–
	2 (*N* = 4)	0.6	E87G	–	–	–
2.5	3 (*N* = 13)	4.8	S83R	–	–	–
	4 (*N* = 7)	4.8	S83R	–	E84K	–

*–: No mutant was found. ^a^*E. coli* numbering. *N*: The number of the samples. 1, 2: The mutant strains that selected from the dose group of 1 mg/kg and the MIC increased to 4.8 and 0.6 mg/L, respectively. 3, 4: The mutant strains that selected from the dose group of 2.5 mg/kg and the MIC increased to 4.8 mg/L.*

## Discussion

*Mycoplasma gallisepticum* not only causes economically significant respiratory disease in chickens and turkeys, but also is a primary pathogen for chukar, red-legged partridges, pheasants and members of the Fringillidae family (Benskin et al., 2009). Quinolones are broad-spectrum antimicrobials and effective in the treatment of *M. gallisepticum* infections (Gerchman et al., 2008; Zhang et al., 2017). The synthetic quinolone danofloxacin is widely used in China for the treatment of chicken respiratory infections caused by *M. gallisepticum.* In order to support it as an antimicrobial chemotherapy with an optimum dose response, *in vivo* model is essential to provide real conditions rather than *in vitro* model. However, quinolones resistance has emerged in *M. gallisepticum* due to improper use of those antimicrobial agents (Pakpinyo and Sasipreeyajan, 2007; Lysnyansky et al., 2008, 2012). In the current study, we strived to establish an *in vivo* infection model and investigate the efficacy of danofloxacin against *M. gallisepticum* using this model. In addition, resistant mutants were selected and preliminary mechanism of danofloxacin resistance was analyzed.

When the inoculum size of *M. gallisepticum* was 8.33 log_10_ CFU/mL, the morbidity, mortality, *M. gallisepticum* load in air sac and lung were consistent with an earlier report (Xiao et al., 2015b). Additionally, for the reason that the reduction of *M. gallisepticum* in air sac and lung was used to fit the PK–PD model by WinNonlin, the appreciative load of *M. gallisepticum* in infection site should be obtained from the *in vivo* infection model. The results showed that the maximum load of *M. gallisepticum* were observed (6.17 log_10_ CFU/air sac and 6.09 log_10_ CFU/lung) when the inoculum size was 9.31 log_10_ CFU/mL. Therefore, it is preferred to apply the inoculum size of 0.2-mL 10^9^ CFU/mL twice daily for 3 days in *in vivo M. gallisepticum* infection model.

In the past decades, there were limited information about the *in vivo* infection model of antibacterial agents against mycoplasmas. This study is the first one to investigate the efficacy of danofloxacin against *M. gallisepticum* in chicken infection model. Comparing with the neutropenic chicken infection model (Xiao et al., 2015b), the main innovative points of this paper are as follows: (1) Not only the drug concentrations in plasma were detected, but also the concentrations in lung tissues were examined; (2) It was the first time to quantitate *M. gallisepticum* colonies in chicken by CFU method that were recorded from inverted microscope directly and the resistant mutants could be selected from the agar plate; (3) The point mutations in QRDR as well as the susceptibility to other quinolones of the selected mutant strains were determined.

In the present investigation, dose proportionality of danofloxacin PK in the range of 1 mg/kg to 20 mg/kg has been described using danofloxacin concentration in plasma and lung tissues in infected chickens. The PK profiles reveal that the average danofloxacin concentration in lung tissues was higher than the corresponding concentration in plasma (1.45–5.14 times higher). According to previously published articles, higher concentrations of danofloxacin in lung tissues than plasma have been reported in other animals as well. The value of peak lung tissues concentration/peak plasma concentration of danofloxacin were 4.5 in sheep (McKellar et al., 1998), 5.0 in pigs (Friis, 1993), and 4.1 in cattle (Giles et al., 1991). The PK of danofloxacin in healthy chickens has been reported by previous studies (Lynch et al., 1994; Knoll et al., 1999; Zeng et al., 2011). Comparing with those studies, there were no significant differences in PK of danofloxacin between the health and infected chickens. For example, *T*_max_ of danofloxacin in health chickens in plasma ranged from 1.5 to 2.33 h; *T*_1/2_ ranged from 6.62 to 13.05 h. However, the *T*_max_ and *T*_1/2_ of danofloxacin in *M. gallisepticum* infected chickens were 2.33 and 11.28 h, respectively. The exception was *C*_max_ in lung of the infected chickens (1.14 μg/mL), which were lower than that of healthy chickens (4.33 μg/mL). Swell was observed in lung of the chickens infected with *M. gallisepticum*. We could speculate that the distribution of danofloxacin in lung may be reduced by these lesions.

Mutations in QRDR of DNA gyrase and topoisomerase IV were the primary quinolone resistance mechanisms for mycoplasma (Bailey et al., 1996; Reinhardt et al., 2002a,b; Gruson et al., 2005; Xie and Zhang, 2006). The substitutions of Ser-83→Arg or Glu-87→Gly in *gyrA* and Glu-84→Lys in *parC* have been described previously (Reinhardt et al., 2002a,b). In a previous report investigated the mechanism of quinolones resistance (Reinhardt et al., 2002a), four different substitutions of *gyrB* in the mutant strains were found. The resistant strains were selected from *in vitro* by incubating in broth medium containing gradually increasing drug concentrations and serial transfers. However, no *gyrB* mutation was observed in the resistant mutant strains selected from *in vivo* infection model. *In vitro* models cannot provide all *in vivo* conditions, because not all conditions for antibacterial agents and bacteria including drug formulation, dosing regimen, sick animals and infecting pathogen have been taken into account in *in vitro* models. Therefore, it is understandable that difference in mutations may be caused by the different infection models.

For quinolones used in both humans and animals, they all exhibit the antibacterial activity by inhibition of bacterial topoisomerase and gyrase, which interfere the replication of DNA (Hopkins et al., 2005). If mutant strains were resistant to one quinolone, resistance to other quinolones were always observed due to the same mechanism of resistance (Hopkins et al., 2005). Our results demonstrate that the danofloxacin-resistant *M. gallisepticum* strains isolated from the infected chickens also conferred the resistance to enrofloxacin, ofloxacin, levofloxacin, gatifloxacin, and norfloxacin. The point mutations in positions 83 and 87 of *gyrA* are the most frequent positions that are the active site of DNA gyrase, which are associated with the replication of DNA during the topoisomerase reaction. The binding of quinolones to the site was altered by the mutations at this active site, resulting in the increased MICs and resistance to quinolones (Willmott and Maxwell, 1993). Our results indicated that MICs of quinolones (danofloxacin, enrofloxacin, ofloxacin, levofloxacin, gatifloxacin, and norfloxacin) against the resistant mutant strains that carried the single mutation in position 83 were higher than that with a single mutation in position 87 of *gyrA*. Similar results were also observed in *Escherichia coli* and *Salmonella* (Ozeki et al., 1997; Bagel et al., 1999; Giraud et al., 1999; Liebana et al., 2002).

In the previous studies (Lysnyansky et al., 2008, 2012), molecular characterization of the QRDRs in enrofloxacin-resistant *M. gallisepticum* strains isolated from various countries was analyzed, revealing that the mutations correlated with the resistant phenotype were the substitutions of Ser-83→Ile or Glu-87→Lys in *gyrA*; Ser-80→Leu in *parC*. However, our results showed that the substitution of Ser83→Arg or Glu-87→Gly in *gyrA*; Glu-84→Lys in *parC* were related to the danofloxacin-resistant *M. gallisepticum* strain S6 isolated from the infected chickens. The difference may be caused by the following reasons: (1) Although the drug and the strains of *M. gallisepticum* were similar, they were not virtually identical; (2) We could speculate that the mutations in clinical isolates were driven by sustained exposure to antimicrobials for many years. These mutant strains survived through a process of natural selection and the mutations could be inherited steadily. However, the mutations in our study were selected under the pressure of a short-term effect of drug, the viability and inheritance of the resistant strains in nature were unknown. Therefore, it is reasonable that the prevalence of QRDR mutations in clinical isolates were different with the strains isolated from laboratory conditions.

## Conclusion

Inoculating with a 0.2-mL aliquot solution containing 10^9^ CFU/mL of the *M. gallisepticum* (twice a day) via intratracheal injection for 3 days was used to establish the ideal *in vivo M. gallisepticum* infection model. In *M. gallisepticum* infected chickens, danofloxacin concentration in lung tissues was higher than plasma. Danofloxacin showed greatly effectiveness against *M. gallisepticum* infection. Mycoplasmacidal activity was found when infected chickens were exposed to danofloxacin at the dose group above 2.5 mg/kg and resistant mutant strains were selected at the dose group of 1 and 2.5 mg/kg. AUC_24_/MIC correlated well with antimycoplasmal efficacy (*R*^2^ = 0.8901). The ratios of AUC_24_/MIC for 2 log_10_ (CFU/mL) reduction and 3 log_10_ (CFU/mL) reduction were 31.97 and 97.98 L⋅h/kg, respectively. Substitutions of Ser-83→Arg or Glu-87→Gly in *gyrA*, Glu-84→Lys in *parC* were detected in the danofloxacin-resistant mutant strains. In addition, the susceptibility of danofloxacin, enrofloxacin, ofloxacin, levofloxacin, gatifloxacin, and norfloxacin against those resistant mutants with a single mutation in position 83 were lower than that with a single mutation in position 87. The results showed that danofloxacin at a dose of 5.5 mg/kg resulted in a reduction of 3 log_10_ CFU/lung.

## Author Contributions

Methodology, software, validation, formal analysis, data curation, writing (original draft preparation), writing (review and editing), visualization, and project administration: NZ. Investigation: NZ, YW, ZH, LY, and LZ; resources: NZ, QC, XS, HJ, and HD. Supervision: HD. Funding acquisition: HD.

## Conflict of Interest Statement

The authors declare that the research was conducted in the absence of any commercial or financial relationships that could be construed as a potential conflict of interest.

## References

[B1] Adler H. E. Fabricant J. Yamamoto R. Berg J. (1958). Symposium on chronic respiratory diseases of poultry. I. Isolation and identification of pleuropneumonia-like organisms of avian origin. *Am. J. Vet. Res.* 19 440–447. 13533769

[B2] Bagel S. Hullen V. Wiedemann B. Heisig P. (1999). Impact of gyrA and parC mutations on quinolone resistance, doubling time, and supercoiling degree of *Escherichia coli*. *Antimicrob. Agents Chemother.* 43 868–875. 10103193 10.1128/aac.43.4.868 PMC89219

[B3] Bailey C. C. Younkins R. Huang W. M. Bott K. F. (1996). Characterization of genes encoding topoisomerase IV of *Mycoplasma genitalium*. *Gene* 168 77–80. 10.1016/0378-1119(95)00718-0 8626069

[B4] Benskin C. M. Wilson K. Jones K. Hartley I. R. (2009). Bacterial pathogens in wild birds: a review of the frequency and effects of infection. *Biol. Rev. Camb. Philos. Soc.* 84 349–373. 10.1111/j.1469-185X.2008.00076.x 19438430

[B5] Friis C. (1993). Penetration of danofloxacin into the respiratory tract tissues and secretions in calves. *Am. J. Vet. Res.* 54 1122–1127. 8396371

[B6] Gerchman I. Lysnyansky I. Perk S. Levisohn S. (2008). In vitro susceptibilities to quinolones in current and archived *Mycoplasma gallisepticum* and *Mycoplasma synoviae* isolates from meat-type turkeys. *Vet. Microbiol.* 131 266–276. 10.1016/j.vetmic.2008.04.006 18534788

[B7] Giles C. J. Magonigle R. A. Grimshaw W. T. Tanner A. C. Risk J. E. Lynch M. J. (1991). Clinical pharmacokinetics of parenterally administered danofloxacin in cattle. *J. Vet. Pharmacol. Ther.* 14 400–410. 10.1111/j.1365-2885.1991.tb00854.x 1663562

[B8] Giraud E. Brisabois A. Martel J. L. Chaslus-Dancla E. (1999). Comparative studies of mutations in animal isolates and experimental in vitro- and in vivo-selected mutants of *Salmonella* spp. suggest a counterselection of highly quinolone-resistant strains in the field. *Antimicrob. Agents Chemother.* 43 2131–2137. 10471553 10.1128/aac.43.9.2131 PMC89435

[B9] Greenberg-Ofrath N. Terespolosky Y. Kahane I. Bar R. (1993). Cyclodextrins as carriers of cholesterol and fatty acids in cultivation of mycoplasmas. *Appl. Environ. Microbiol.* 59 547–551. 8434920 10.1128/aem.59.2.547-551.1993 PMC202141

[B10] Gruson D. Pereyre S. Renaudin H. Charron A. Bebear C. Bebear C. M. (2005). In vitro development of resistance to six and four quinolones in *Mycoplasma pneumoniae* and *Mycoplasma hominis*, respectively. *Antimicrob. Agents Chemother.* 49 1190–1193. 10.1128/AAC.49.3.1190-1193.2005 15728924 PMC549269

[B11] Hannan P. C. (2000). Guidelines and recommendations for antimicrobial minimum inhibitory concentration (MIC) testing against veterinary mycoplasma species. International Research Programme on Comparative Mycoplasmology. *Vet. Res.* 31 373–395. 10.1051/vetres:2000100 10958240

[B12] Hopkins K. L. Davies R. H. Threlfall E. J. (2005). Mechanisms of quinolone resistance in *Escherichia coli* and *Salmonella*: recent developments. *Int. J. Antimicrob. Agents* 25 358–373. 10.1016/j.ijantimicag.2005.02.006 15848289

[B13] Jordan F. T. Horrocks B. K. Jones S. K. Cooper A. C. Giles C. J. (1993). A comparison of the efficacy of danofloxacin and tylosin in the control of *Mycoplasma gallisepticum* infection in broiler chicks. *J. Vet. Pharmacol. Ther.* 16 79–86. 10.1111/j.1365-2885.1993.tb00292.x 8386777

[B14] Kleven S. (1990). Summary of discussions of avain mycoplasma team international researh program on comparative mycoplasmology babolna, Hungary. *Avian Pathol.* 19 795–800. 10.1080/03079459008418732

[B15] Knoll U. Glunder G. Kietzmann M. (1999). Comparative study of the plasma pharmacokinetics and tissue concentrations of danofloxacin and enrofloxacin in broiler chikens. *J. Vet. Pharmacol. Ther.* 22 239–246. 10.1046/j.1365-2885.1999.00217.x 10499235

[B16] Levisohn S. Kleven S. (2000). Avian mycoplasmosis (*Mycoplasma gallisepticum*). *Rev. Sci. Tech.* 19 425–442. 10.20506/rst.19.2.1232 10935272

[B17] Ley D. H. Berkhoff J. E. Levisohn S. (1997). Molecular epidemiologic investigations of *Mycoplasma gallisepticum* conjunctivitis in songbirds by random amplified polymorphic DNA analyses. *Emerg. Infect. Dis* 3 375 10.3201/eid0303.970318 PMC2627637 9284386

[B18] Liebana E. Clouting C. Cassar C. A. Randall L. P. Walker R. A. Threlfall E. J. (2002). Comparison of gyrA mutations, cyclohexane resistance, and the presence of class I integrons in *Salmonella enterica* from farm animals in England and Wales. *J. Clin. Microbiol.* 40 1481–1486. 10.1128/JCM.40.4.1481-1486.2002 11923377 PMC140356

[B19] Lynch M. J. Rice J. R. Ericson J. F. Mosher F. R. Millas W. J. Harran L. P. (1994). Residue depletion studies on danofloxacin in the chicken. *J. Agric. Food Chem.* 42 289–294. 10.1021/jf00038a012

[B20] Lysnyansky I. Gerchman I. Levisohn S. Mikula I. Feberwee A. Ferguson N. M. (2012). Discrepancy between minimal inhibitory concentration to enrofloxacin and mutations present in the quinolone-resistance determining regions of *Mycoplasma gallisepticum* field strains. *Vet. Microbiol.* 160 222–226. 10.1016/j.vetmic.2012.05.002 22655973

[B21] Lysnyansky I. Gerchman I. Perk S. Levisohn S. (2008). Molecular characterization and typing of enrofloxacin-resistant clinical isolates of *Mycoplasma gallisepticum*. *Avian Dis.* 52 685–689. 10.1637/8386-063008-RESNOTE.1 19166064

[B22] McKellar Q. A. Gibson I. F. McCormack R. Z. (1998). Pharmacokinetics and tissue disposition of danofloxacin in sheep. *Biopharm. Drug Dispos.* 19 123–129. 10.1002/(SICI)1099-081X(199803)19:2<123::AID-BDD89>3.0.CO;2-G 9533113

[B23] Ozeki S. Deguchi T. Yasuda M. Nakano M. Kawamura T. Nishino Y. (1997). Development of a rapid assay for detecting gyrA mutations in *Escherichia coli* and determination of incidence of gyrA mutations in clinical strains isolated from patients with complicated urinary tract infections. *J. Clin. Microbiol.* 35 2315–2319. 9276409 10.1128/jcm.35.9.2315-2319.1997 PMC229961

[B24] Pakpinyo S. Sasipreeyajan J. (2007). Molecular characterization and determination of antimicrobial resistance of *Mycoplasma gallisepticum* isolated from chickens. *Vet. Microbiol.* 125 59–65. 10.1016/j.vetmic.2007.05.011 17570621

[B25] Razin S. Tully J. G. (1970). Cholesterol requirement of mycoplasmas. *J. Bacteriol.* 102 306–310. 4911537 10.1128/jb.102.2.306-310.1970 PMC247552

[B26] Reinhardt A. K. Bébéar C. Kobisch M. Kempf I. Gautier-Bouchardon A. (2002a). Characterization of mutations in DNA gyrase and topoisomerase IV involved in quinolone resistance of *Mycoplasma gallisepticum* mutants obtained in vitro. *Antimicrob. Agents Chemother.* 46 590–593. 11796386 10.1128/AAC.46.2.590-593.2002 PMC127038

[B27] Reinhardt A. K. Kempf I. Kobisch M. Gautier-Bouchardon A. V. (2002b). Quinolone resistance in *Mycoplasma gallisepticum*: DNA gyrase as primary target of enrofloxacin and impact of mutations in topoisomerases on resistance level. *J. Antimicrob. Chemother.* 50 589–592. 12356806 10.1093/jac/dkf158

[B28] Toutain P. L. Bousquet-Melou A. Martinez M. (2007). AUC/MIC: a PK/PD index for antibiotics with a time dimension or simply a dimensionless scoring factor? *J. Antimicrob. Chemother.* 60 1185–1188. 10.1093/jac/dkm360 17932075

[B29] Vinks A. Derendorf H. Mouton J. (2014). *Fundamentals of Antimicrobial Pharmacokinetics and Pharmacodynamics.* Berlin: Springer.

[B30] Willmott C. J. Maxwell A. (1993). A single point mutation in the DNA gyrase A protein greatly reduces binding of quinolones to the gyrase-DNA complex. *Antimicrob. Agents Chemother.* 37 126–127. 10.1128/AAC.37.1.126 8381633 PMC187618

[B31] Xiao X. Sun J. Chen Y. Zou M. Zhao D.-H. Liu Y.-H. (2015a). Ex vivo pharmacokinetic and pharmacodynamic analysis of valnemulin against *Mycoplasma gallisepticum* S6 in *Mycoplasma gallisepticum* and *Escherichia coli* co-infected chickens. *Vet. J.* 204 54–59. 10.1016/j.tvjl.2015.01.020 25744809

[B32] Xiao X. Sun J. Yang T. Fang X. Wu D. Xiong Y. Q. (2015b). In Vivo pharmacokinetic/pharmacodynamic profiles of valnemulin in an experimental intratracheal *Mycoplasma gallisepticum* infection model. *Antimicrob. Agents Chemother.* 59 3754–3760. 10.1128/AAC.00200-15 25845865 PMC4468724

[B33] Xie X. Zhang J. (2006). Trends in the rates of resistance of *Ureaplasma urealyticum* to antibiotics and identification of the mutation site in the quinolone resistance-determining region in Chinese patients. *FEMS Microbiol. Lett.* 259 181–186. 10.1111/j.1574-6968.2006.00239.x 16734777

[B34] Zeng Z. Deng G. Shen X. Zeng D. Ding H. (2011). Plasma and tissue pharmacokinetics of danofloxacin in healthy and in experimentally infected chickens with *Pasteurella multocida*. *J. Vet. Pharmacol. Ther.* 34 101–104. 10.1111/j.1365-2885.2010.01223.x 21219355

[B35] Zhang N. Gu X. Ye X. Wu X. Zhang B. Zhang L. (2016). The PK/PD interactions of doxycycline against *Mycoplasma gallisepticum*. *Front. Microbiol.* 7:653 10.3389/fmicb.2016.00653 PMC4854994 27199972

[B36] Zhang N. Ye X. Wu Y. Huang Z. Gu X. Cai Q. (2017). Determination of the mutant selection window and evaluation of the killing of *Mycoplasma gallisepticum* by Danofloxacin, Doxycycline, Tilmicosin, Tylvalosin and Valnemulin. *PLoS ONE* 12:e0169134 10.1371/journal.pone.0169134 PMC5215565 28052123

